# Neighborhood social cohesion and physical disorder in relation to social isolation in older adults: racial and ethnic differences

**DOI:** 10.1186/s12889-024-20112-9

**Published:** 2024-09-20

**Authors:** Weidi Qin, Emily J. Nicklett, Jiao Yu, Ann W. Nguyen

**Affiliations:** 1 Sandra Rosenbaum School of Social Work, University of Wisconsin–Madison, 1350 University Ave, Madison, WI 53706 USA; 2 College for Health, Community and Policy, University of Texas at San Antonio, San Antonio, TX USA; 3 School of Public Health, Yale University, New Haven, CT USA; 4 Jack, Joseph and Morton Mandel School of Applied Social Sciences, Case Western Reserve University, Cleveland, OH USA

**Keywords:** Social isolation, Neighborhood context, Minority health, Older adults

## Abstract

**Background:**

Neighborhood factors of social isolation have been understudied, hindering efforts to reduce social isolation at the neighborhood level. This study aims to investigate the longitudinal effects of neighborhood social cohesion and physical disorder on social isolation in community-dwelling older adults, as well as to examine whether race/ethnicity moderates the neighborhood-isolation relationship.

**Methods:**

We used 11-year data from the National Health and Aging Trend Study, a longitudinal national study of Medicare beneficiaries aged 65 and older. Social isolation was measured through a summary score across four domains: marital/partner status, family and friend contact, religious attendance, and club participation. A series of weighted mixed-effects logistic regression models were performed to test the study aims. Sample sizes ranged from 7,303 to 7,291 across individual domains of social isolation.

**Results:**

Approximately 20% of participants reported social isolation. Findings indicated a negative association between neighborhood social cohesion and social isolation. Higher levels of neighborhood social cohesion were longitudinally associated with lower odds of social isolation (odds ratio [OR] = 0.52, 95% CI: 0.47–0.58). Yet, the presence of neighborhood physical disorder was associated with an increased risk of overall social isolation ([OR] = 1.2, 95% CI: 1.00, 1.44). Race/ethnicity significantly moderated the effects of neighborhood social cohesion and physical disorder on social isolation. The odds of no in-person visits associated with neighborhood social cohesion are smaller among Black adults compared to White adults. Black adults had constantly lower odds of isolation from religious attendance compared to White adults regardless of the level of neighborhood social cohesion. Hispanic adults had decreased odds of having no friends associated with signs of physical disorder, while no associations were found among older White adults. White adults had higher odds of isolation from in-person visits when living in neighborhoods with signs of physical disorder, whereas no association was observed among older Black and Hispanic adults.

**Conclusions:**

This study elucidates the role of neighborhood characteristics in shaping social isolation dynamics among older adults. Furthermore, the observed moderation effects of race/ethnicity suggest the need for culturally sensitive interventions tailored to address social isolation within specific neighborhood and racial contexts.

**Supplementary Information:**

The online version contains supplementary material available at 10.1186/s12889-024-20112-9.

## Introduction

Social isolation, defined as a paucity of social network members and social contact with family and friends [1], affects approximately 34–56% of older adults aged 65 years and over [2]. Experiences of social isolation can occur when older adults experience reduced contact with family and friends (such as when same-age family members and friends die), decreased religious attendance, and withdrawal from social activities [1]. Social isolation has significant effects on late-life well-being, as older adults are at heightened risk of experiencing life events such as death of spouse, retirement, and restrictions in social participation due to health limitations [3]. These late-life events may reduce older adults’ opportunities to engage in meaningful interactions with the network members. Prolonged social isolation is associated with adverse health and social consequences. For example, social isolation is associated with increased mortality [4], dementia risk [5], and poor mental health [6]. Furthermore, social isolation is costly as it accounts for an estimated annual $6.7 billion in excess of Medicare spending among older adults [7]. Taken together, social isolation represents an urgent public health issue and a major social risk factor of late-life well-being.

Determinants of social isolation among older adults have been extensively studied. Prior evidence suggests that common late-life events, such as driving cessation, disability, hospitalization, and smaller social network size are associated with increased risk of social isolation [8–10]. Existing research on contributing factors of social isolation has heavily emphasized individual-level factors, which has informed the development of intervention strategies aimed at mitigating social isolation. For example, a recent systematic review of interventions targeting social isolation among older adults indicates that existing intervention points are largely focused on individual-level factors such as promoting social or physical activities, providing counseling and therapy, and offering internet training and home service provision [11]. While individual-level factors are crucial in addressing social isolation, there is a noticeable gap in the literature when it comes to exploring neighborhood-level factors contributing to the elevated risk of social isolation. Further investigation of the contextual risks of social isolation can advance knowledge about pathways linking contextual factors to late-life health and can inform community-level initiatives to reduce social isolation among older adults. Moreover, a more in-depth understanding of the role that neighborhood characteristics play in social isolation can also contribute significantly to existing knowledge of how environmental factors and social ties impact late-life well-being.

Neighborhood characteristics are important social determinants of health. The Social Disorganization Theory, originally developed to understand crime in the neighborhoods, argues that perceptions of neighborhood social and physical conditions can induce stress [12]. Informed by the Social Disorganization Theory, two aspects of social processes within the neighborhoods were developed, which are *social cohesion* (i.e., relational characteristics and perceived collective efficacy of one’s neighborhood) and *physical disorder* (i.e., visible signs of physical decay and deterioration in one’s neighborhood) [13, 14]. Social disorganization theory also provides a useful framework for examining racial and ethnic differences in social isolation within neighborhood context. Contemporary elaborations also suggest that the lack of collective efficacy in the neighborhoods may contribute to adverse psychosocial experiences, especially among Black individuals who are more likely to reside in under-resourced communities [15, 16].

### Perceived social cohesion

Neighborhood social cohesion characterizes the collective support and solidarity within a neighborhood and can impact health outcomes at the individual level [17, 18]. Positive perceptions of the neighborhood social cohesion may be indicative of supportive interactions and trusting networks with neighbors, which can protect against social isolation and loneliness among older adults [19, 20]. Earlier research shows that low social cohesion is associated with reduced social participation, such as visiting friends and family, participating in social activities, and going out for enjoyment [21], pointing to a potential pathway linking neighborhood social cohesion to social isolation among older adults living in the community. Older adults living in neighborhoods characterized by low social cohesion may lack a sense of community and are unlikely to experience supportive relationships with their neighbors, leading to reduced social contacts and support networks. In related research, earlier evidence suggests that higher levels of neighborhood social cohesion is linked to stronger perceived companionship among community-dwelling older adults, especially among those who live alone [22]. Older adults are at higher risk of social isolation due to life changes such as retirement and health declines, and neighborhood with a strong perceived social cohesion can reflect an important source of social interactions that can prevent social isolation. Overall, it is possible that neighborhoods with strong social cohesion can provide opportunities for positive social interactions and reduce social isolation among older adults.

### Perceived physical disorder

On the other hand, neighborhood physical disorder signals declining physical neighborhood environments that can have adverse effects on health [12, 14]. Perceived disadvantages in the neighborhood can deter older adults from going outside and engaging in social interactions with their neighborhood networks due to fear of unsafe environment and crime [21], thus increasing the risk of social isolation. Earlier findings show that perceived neighborhood deterioration, such as physical conditions of buildings in the neighborhood and safety from crime, are related to increased social isolation among older adults, especially among those with lower levels of educational attainment [23]. Negative perceptions of neighborhood physical conditions may indicate mistrust among older adults, who tend to self-isolate and decline social support in the face of mistrust [24]. Taken together, older residents living in neighborhood with declining physical features, such as extensive littering and vacant buildings, may tend to avoid interactions with others in their community and experience isolation. Additional evidence from related research also indicates that neighborhood physical disorder is associated with higher odds of dementia onset via indirect effects on subjective social isolation [25], emphasizing the role of neighborhood deprivation in late-life psychosocial well-being.

### Longitudinal data

Existing research examining neighborhood characteristics and social isolation among older adults are largely limited to cross-sectional or two-wave findings [19, 21, 26]. A new study using a longitudinal design shows that living in more cohesive neighborhoods is linked to reduced levels of loneliness among older adults, but the effects of physical disorder in the neighborhood remain unaddressed [27]. Given that most older adults live in the community and are likely to be influenced by their immediate social and physical environment in the neighborhood, additional investigation is warranted to quantify the long-term effects of both social and physical neighborhood factors on social isolation.

### Racial and ethnic differences

Race and ethnicity could moderate the role of the neighborhood context on social isolation among older adult populations. Some studies have found that older White adults are more likely than older Black and Hispanic adults to experience social isolation in adjusted analyses [28]. However, other studies have found that Black adults experience more social isolation and less social engagement, overall, than White adults and people of other races, while Hispanic adults experience much less social isolation than White adults [29]. One potential driver of these conflicting findings relates to the multifaceted nature of the construct of social isolation. Compared to Black adults, White adults are more likely to live alone, to be childless, and to have limited contact with religious congregations [30]. With the absence of social connection in these domains, White individuals may seek connections elsewhere, including friends and clubs/associations in their local neighborhood communities.

The social and physical resources available to community members differ substantially by neighborhood, and by who resides in those neighborhoods. Prior studies of older adults have found that perceived neighborhood social cohesion is highest—and perceived neighborhood social disorder is lowest—among White participants, followed by Hispanic participants, then Black participants [31]. The racial and ethnic disparities in the neighborhood physical environment are a consequence of the historical and ongoing impact of policies embedded in structural racism, such as redlining and other forms of racial residential segregation. Consequently, compared to neighborhoods where residents are predominantly Hispanic or Black, predominantly White neighborhoods are less likely to show signs of physical disorder [32, 33] and are more likely to have access to blue/green spaces [33, 34], evenly constructed sidewalks [35, 36], and other amenities that facilitate social interaction and social connection in local community settings [37]. Older adults who reside in communities with greater amenities, and with fewer environmental barriers, have greater opportunities to seek and nurture social connections within those communities.

Older Black and Hispanic adults may possess more risk factors of social isolation compared to their White peers. One such risk factor could be adverse experiences in the neighborhood. For example, older Black and Hispanic adults are more likely to report negative perceptions of their neighborhood environment, such as low social cohesion and high physical disorder [38], suggesting that older Black and Hispanic adults are more likely to reside in stress-inducing neighborhoods partly due to historical segregation. However, the effects of negative neighborhood perceptions on social isolation are unclear. For example, a recent within-group study among older Black adults suggests that neighborhood perceptions have limited influence on social isolation among older Black Americans [26]. On the contrary, related research among Black families shows that neighborhood disadvantage (i.e., concentrated poverty) is linked to increased risk of social isolation, such as smaller network size and less organizational participation [39]. Similarly, racial differences were found in the neighborhood effects on adverse functional and cognitive outcomes [38, 40], suggesting that neighborhood characteristics may operate differently across racial and ethnic groups. However, few studies have examined the moderating effects of race and ethnicity on neighborhood characteristics and social isolation, hindering efforts to develop culturally specific interventions within the neighborhood environment.

To bridge the research gaps, the present study aims to utilize 11-year longitudinal data to address the following two research questions: (1) Are neighborhood characteristics longitudinally associated with social isolation among community-dwelling older adults? and (2) Does race and ethnicity moderate the association between neighborhood characteristics on social isolation over time? We hypothesize that (1) a lower level of social cohesion and a greater level of physical disorder in the neighborhood are associated with increased social isolation over time, and (2) race longitudinally moderates the neighborhood-isolation association. That is, the magnitudes of associations between neighborhood characteristics and social isolation specified in Hypothesis 1 are larger among older Black and Hispanic adults than Older White adults.

## Methods

### Study sample

Data came from Round 1 (2011) to Round 11 (2021) of the National Health and Aging Trend Study (NHATS). NHATS is a longitudinal panel study conducted annually among a nationally representative sample of Medicare beneficiaries aged 65 or older [41]. NHATS respondents were recruited from the Medicare enrollment database through a stratified three-stage sample design [41]. NHATS features an oversampling of individuals aged 85 or older and older Black adults. The rich data on psychosocial indicators made NHATS ideal to examine the present study aims. For Round 1, 12,411 cases were sampled and 8,245 individuals responded, resulting in a response rate of 71% [41]. Excluding the nursing home residents, the baseline sample size of community-dwelling older adults was *N* = 7,609, who were followed up and surveyed annually.

### Measures

***Social Isolation***. We used a validated index of social isolation developed based on measures in the NHATS [1]. Specifically, six items from the NHATS were used to create a summary score of social isolation, representing four domains (marital/partner status, family/friends contact, religious participation, and club participation) of social integration and network [42]. Specifically, a point of 1 was assigned for each of the following situations: (1) not married or living with a partner; (2) unable to identify a family member with whom respondents talked most often about important things over the last year; (3) unable to identify a friend with whom respondents talked most often about important things over the last year; (4) no in-person visits with family or friends in the last month; (5) no attendance at religious services in the last month, and (6) no participation in clubs, classes, or other organized activities in the last month. The points were summed up to form a summary score of social isolation that measured the deficiency of social contacts and integrating relationships. The summary score ranged from 0 to 6 with a higher score indicating greater social isolation. Previous research suggested cut-off points to classify the social isolation status [1, 5]. Specifically, individuals with a score of 4 or more were considered socially isolated and those with a score below 4 were considered not isolated [5]. Social isolation is a time-varying variable that was measured at each round of data collection.

***Neighborhood Characteristics***. Measures of neighborhood social cohesion and physical disorder were adapted from previously validated scales developed based on samples of two population-based cohort studies of older adults [13]. The two scales have been extensively utilized in aging research [21, 43]. Neighborhood social cohesion was measured with a 3-item scale that asked respondents to what extent they agreed with the following statements: In the community where you live, (1) people know each other well; (2) people are willing to help each other; and (3) people can be trusted. The response was documented on a 3-point scale: do not agree (1), agree a little (2) and agree a lot (3). A total summary score was created by averaging across the scale items, ranging from 1 to 3, with a higher score indicating more positive perceptions of neighborhood social cohesion and community support (Cronbach’s α = 0.73).

Neighborhood physical disorder was measured using a 3-item environmental checklist, which was completed by the NHATS interviewers based on their observations on the neighborhood area around the respondents’ residence. Specifically, the interviewers documented how much they observed the following: (1) litter or trash on the ground; (2) graffiti on walls; (3) vacant homes or stores. The response categories were on a 4-point scale: none (1), a little (2), some (3), a lot (4). The total score was created by averaging across the scale items, with a higher score indicating greater physical disorder in the community (Cronbach’s α = 0.73). Because the neighborhood physical disorder was highly skewed (skewness = 4.67; Kurtosis = 31.72) and only a small percentage of any sign of physical disorder was observed (about 14%), we dichotomized the scale to indicate no physical disorder (score = 0) and any signs of physical disorder (score > 0). By using respondent-reported social cohesion and interviewer-observed physical disorder in the neighborhood, we were able to assess both social and physical aspects of the neighborhood environment. The two measures on neighborhood characteristics are time-varying variables that were measured at each round of data collection.

***Covariates***. The selection of covariates was based on previous research on social isolation among older adults [6, 8, 26]. Socio-demographic factors and health indicators that may be associated with social isolation were included as covariates to allow for estimation of the independent effects of neighborhood. Socio-demographic controls included age (in years), gender (male or female), race/ethnicity (white, black, Hispanic, others), and educational attainment (less than high school, high school graduate or GED, some college but no degree, college degree or above). Dementia status was controlled due to its association with social isolation [5]. In NHATS, dementia status was classified into three categories (no dementia, possible dementia, or probable dementia) based on a series of assessments including cognitive tests, AD8 Screening Interview, and self-reported diagnosis [44]. The count of chronic conditions was calculated as a sum of eight self-reported conditions (high blood pressure, diabetes, heart disease or heart attack, arthritis, osteoporosis, lung disease, cancer, and stroke). Depressive symptoms status was measured with a two-item Patient Health Questionnaire: Over the last month, how often have you (1) had little interest or pleasure in doing things, and (2) felt down, depressed, or hopeless? A score of 3 or greater was defined as having depressive symptoms [45]. Receiving caregiving (Yes or No) was ascertained by whether older adults received help with any of the nine activities (eating, bathing, using the toilet, dressing, doing laundry, grocery shopping, meal preparation, banking, taking medication, going outside home, getting around inside home, and getting out of bed) [46]. Proxy respondents were included and controlled to reduce the selection bias and avoid reduced sample size [47].

### Data analysis

Baseline sample characteristics were described for the full sample and compared between individuals who were isolated and not isolated using chi-square tests or t-tests. We also compared differences in neighborhood factors and individual domains of social isolation by race and ethnicity, using analysis of variance (ANOVA) and Chi-squared tests. Post-hoc analyses using the Bonferroni correction were conducted to test pairwise differences between racial and ethnic groups. A series of mixed-effects logistic regression models were performed in two steps. We adopted a complete case analysis (CCA) approach in performing mixed-effects models [48]. The mixed-effects model does not assume the same number of timepoints for each participant. That is, individual participants who have missing data at a given interview wave were not excluded [49]. We used predictors (i.e., neighborhood factors) in Round t to predict social isolation outcomes in the same round (Round t). In step 1, we examined the main effects of neighborhood characteristics on overall social isolation and each domain of social isolation, controlling for socio-demographic and health covariates. In step 2, two interaction terms, “social cohesion × race” and “physical disorder × race”, were created and added to models in Step 1 to examine the moderating effects of race in neighborhood characteristics and social isolation. We used NHATS-provided sampling weights and survey design factors (i.e., cluster, and strata) were utilized to calculate weights at the levels of survey wave (level 1) and respondent (level 2), and were incorporated in mixed-effects linear regression models to generate weighted estimates [48]. We did not utilize a cross-lagged design and adjust for social isolation at time t-1 as a covariate because our study did not aim to test the reciprocal causal relationship between neighborhood factors and social isolation. To evaluate the robustness of our study findings, sensitivity analyses were conducted by including only non-proxy respondents and by using a 1-year lagged predictor variables (i.e., neighborhood factors in Round t-1 predicting social isolation in Round t). All analyses were conducted in STATA/SE 18 [50].

## Results

Baseline sample characteristics for the entire sample and stratified by isolation status are presented in Table 1. Overall, the study respondents had an average age of 75 years (95% confidence interval [CI]: 75.3–75.7). The majority were female (57%) and White adults (81%). About one in five of the sample had an educational attainment level less than high school (22%), and one in ten had probable dementia (10%) and possible dementia (11%). The mean count of chronic conditions was 2.4 (95% CI: 2.3–2.4). On average, respondents scored 2.4 on the neighborhood social cohesion scale (95% CI: 2.3–2.4). About 11% of respondents lived in neighborhoods with the presence of physical disorder as observed by the NHATS interviewers.

**Table 1:** Sample characteristics by status of isolation at baseline

	All	Not Isolated	Isolated	Range
Age (mean)	75.5 (75.3, 75.7)	74.6 (74.4, 74.8)	78.0 (77.4, 78.5)	65–106
Gender (%)				
Male	42.9 (41.5, 44.3)	43.9 (42.3, 45.5)	41.4 (38.9, 44.0)	
Female	57.1 (55.7, 58.5)	56.1 (54.5, 57.7)	58.6 (56.0, 61.1)	
Race/ethnicity (%)				
Non-Hispanic White	81.4 (79.6, 83.5)	83.6 (83.0, 85.1)	72.8 (69.2, 76.2)	
Non-Hispanic Black	8.3 (7.5, 9.2)	7.2 (6.4, 8.0)	12.0 (10.7, 13.5)	
Hispanic	6.7 (5.7, 7.9)	6.0 (5.1, 7.1)	9.8 (7.6, 12.5)	
Other	3.5 (2.8, 4.5)	3.1 (2.4, 4.0)	5.3 (3.8, 7.3)	
Educational level (%)				
Less than high school	21.8 (20.1, 23.6)	17.6 (16.1, 19.2)	38.1 (35.1, 41.2)	
High school graduate	27.6 (26.3, 28.9)	27.1 (25.6, 28.7)	29.5 (27.5, 31.6)	
Some college, no degree	21.4 (20.3, 22.6)	22.2 (21.0, 23.5)	18.2 (16.0, 20.8)	
College graduate or above	29.2 (26.9, 31.6)	33.1 (30.5, 35.8)	14.1 (12.2, 16.2)	
Dementia status (%)				
No dementia	79.0 (77.4, 80.6)	84.2 (82.7, 85.6)	59.0 (55.9, 62.3)	
Possible dementia	10.9 (9.7, 12.2)	10.0 (8.8, 11.4)	14.4 (12.4, 16.6)	
Probable dementia	10.0 (9.3, 10.8)	5.8 (5.2, 6.4)	2.7 (2.4, 2.9)	
Count of chronic conditions (mean)	2.4 (2.3, 2.4)	2.3 (2.3, 2.4)	2.7 (2.6, 2.8)	0–8
Depressive symptoms (%)	14.6 (13.4, 15.8)	12.1 (10.9, 13.4)	24.2 (22.2, 26.4)	
Receiving caregiving (%)				
No	73.3 (72.2, 74.4)	79.2 (78.0, 80.4)	50.4 (47.4, 53.4)	
Yes	26.7 (25.6, 27.8)	20.8 (19.6, 22.0)	49.6 (46.6, 52.6)	
Neighborhood social cohesion (mean)	2.4 (2.3, 2.4)	2.4 (2.4, 2.5)	2.3 (2.2, 2.3)	1–3
Neighborhood physical disorder (%)	10.9 (9.6, 12.4)	9.1 (7.9, 10.5)	18.0 (15.6, 20.7)	
Proxy respondents (%)	5.8 (4.2, 6.5)	1.7 (1.4, 2.1)	21.9 (19.4, 24.6)	
Sample Size	7,609	5,747	1,862	
Estimated proportion in the population (%)	–	79.5 (78.2, 80.8)	20.5 (19.2, 21.8)	

Note. Means and percents were adjusted for NHATS-provided survey weights and design factors (strata and cluster). 95% confidence intervals were presented in brackets. Range was presented for continuous variables. All comparisons were statistically significant at *p* < 0.001

The estimated proportion of social isolation in the older adult population was about 20%. Baseline sample characteristics stratified by isolation status were all statistically different at *p* < 0.05. In bivariate analyses, study respondents who were categorized as socially isolated were more likely to be older, female, Black and Hispanic individuals, and were less likely to have completed high school. In terms of health covariates, study respondents who had possible dementia, a greater number of chronic conditions, and elevated depressive symptoms were more likely to be classified as isolated. For neighborhood characteristics, older adults who lived in neighborhoods with the presence of physical disorder and lower levels of social cohesion were at higher risk of being isolated.

Table 2 presents the descriptive statistics for the subitems of the social isolation scale. Overall, the most frequent item is no religious attendance in the past month (43%), followed by being unmarried or unpartnered (43%). The least frequent isolation item is no in-person visit with family or friends (12%) followed by no family member to talk to about important things (17%). Among respondents who were classified as socially isolated, the most frequent isolation domain was no club participation (95%) followed by having no friends to talk to (90%), whereas the least frequent domain was no in-person visit to family and friends (42%) followed by no family to talk to (49%). Table 3 presents differences in neighborhood factors and social isolation domains stratified by race and ethnicity. Results showed that White respondents reported higher cohesion than both Black and Hispanic respondents (*p* < 0.001). Higher cohesion was also perceived by Black respondents compared to Hispanic individuals (*p* < 0.01).

**Table 2:** Percents of social isolation domains by overall isolation status at baseline

Isolation items (%)	Overall Sample	Not Isolated	Isolated
Unmarried/unpartnered	43.0 (41.6, 44.4)	33.9 (32.6, 35.2)	78.6 (76.2, 80.7)
No family to talk to	17.5 (16.4, 18.7)	9.5 (8.6, 10.5)	48.8 (45.5, 52.2)
No friend to talk to	75.5 (74.2, 76.9)	71.4 (69.7, 73.0)	90.4 (88.4, 92.0)
No in-person visits with family and friends	12.5 (11.3, 13.9)	4.9 (4.1, 5.8)	42.3 (39.3, 45.4)
No religious attendance	43.2 (41.3, 45.1)	32.8 (30.8, 34.8)	83.8 (81.8, 85.6)
No club participation	62.6 (60.7, 64.6)	54.3 (52.2, 56.4)	94.9 (93.3, 96.1)

Note. Percents were adjusted for complex survey weights and design factors (strata and cluster). 95% confidence intervals presented in brackets. All comparisons were statistically significant at *p* < 0.001

**Table 3:** Baseline neighborhood factors and social isolation by race and ethnicity

	Overall	White (A)	Black (B)	Hispanic (C)	*p* value
**Neighborhood Factors**					
Perceived neighborhood Social Cohesion (mean, SD)	2.4 (0.6)	2.5 (0.5)	2.3 (0.6)	2.2 (0.6)	AB*** AC*** BC**
Perceived Presence of neighborhood Physical Disorder (%)	13.8	7.8	28.8	25.8	AB*** AC***
**Social Isolation**					
Unmarried/unpartnered (%)	50.0	45.5	64.4	51.9	AB*** BC***
No family to talk to (%)	19.8	16.8	26.3	22.4	AB*** AC*
No friend to talk to (%)	77.4	76.2	77.1	88.8	AC*** BC***
No in-person visits with family and friends (%)	14.7	11.9	19.8	24.2	AB*** AC***
No religious attendance (%)	42.0	44.6	33.0	43.0	AB*** BC**
No club participation (%)	65.6	61.6	73.1	81.1	AB*** AC*** BC**

Note. ANOVA was used to test differences in neighborhood social cohesion by race and ethnicity. Chi-square was used to test differences in the presence of neighborhood physical disorder by race and ethnicity. The Bonferroni correction was used to test pairwise differences between each racial and ethnic groups

*p < 0.05, **p < 0.01, ***p < 0.001

Conversely, the presence of perceived neighborhood physical disorder was significantly lower among White respondents compared to Black and Hispanic respondents (*p* < 0.001), but no difference was observed between Black and Hispanic respondents. In terms of social isolation domains, White respondents reported less isolation than Black respondents in the domains of being unmarried, no family to talk to, no in-person visit, and no club participation. However, White adults were more likely to report no religious attendance than both Black and Hispanic respondents.

Supplementary Figs. 1–3 show the trend of changes in neighborhood social cohesion, physical disorder, and social isolation over 11 years. The level of perceived physical disorder had a declining trend but increased from 2019 to 2021. The level of social isolation increased from 2019 to 2020 and then dropped in 2021.

### Hypothesis 1

**Fig. 1:**
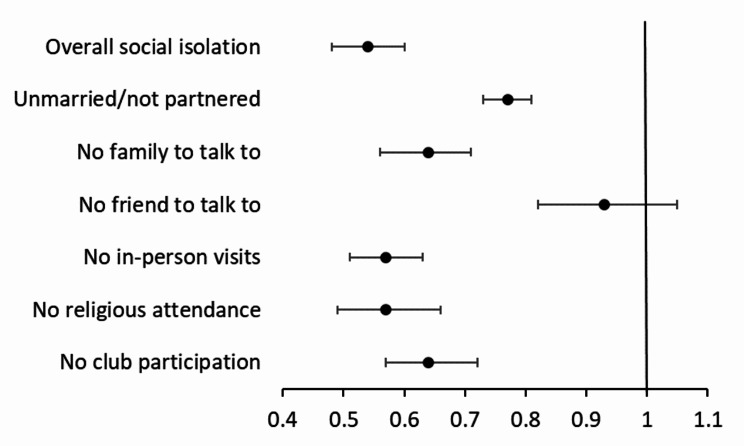
Results from mixed-effects logistic models of neighborhood social cohesion predicting overall social isolation and each domain of isolation, adjusting for socio-demographic, health controls, and receiving caregiving. Results are based on Supplementary Table 1

**Fig. 2:**
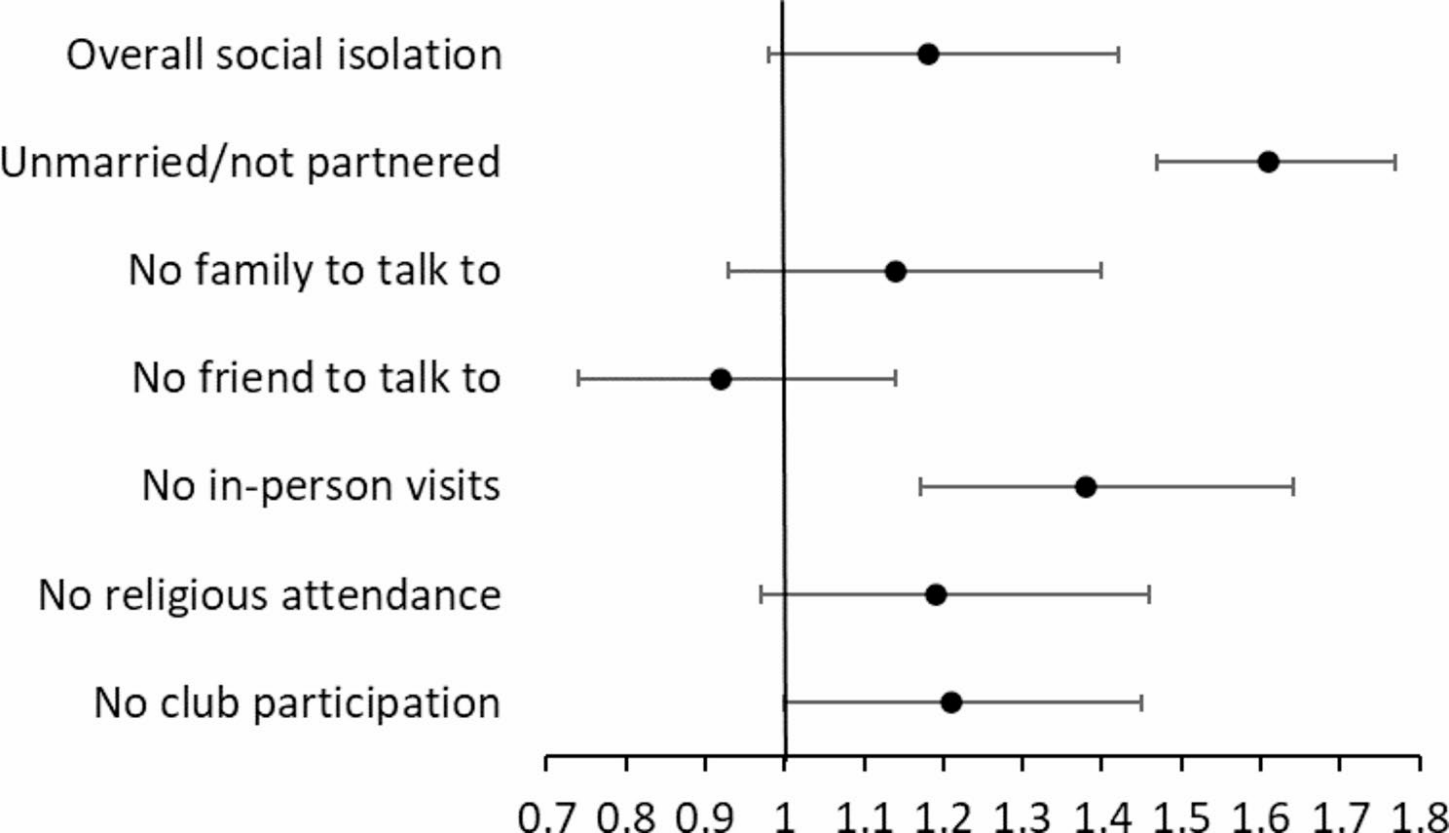
Results from multivariable mixed-effects logistic models of neighborhood physical disorder predicting overall social isolation and each domain of isolation, adjusting for socio-demographic, health controls, and receiving caregiving. Results are based on Supplementary Table 1

Results from the multivariable mixed-effects logistic regressions of longitudinal associations between neighborhood characteristics and social isolation (overall and individual domains) are plotted in Fig. 1 (social cohesion) and Fig. 2 (physical disorder) based on Supplementary Table 1. Controlling for socio-demographic and health covariates, a greater level of neighborhood social cohesion was longitudinally associated with lower odds of social isolation and each individual domain except having no friend to talk to about important things. Over time, a one-unit increase in the level of neighborhood social cohesion was related to about 56% lower odds of overall social isolation (odds ratio [OR] = 0.54, 95% CI: 0.48–0.60), 23% lower odds of being unmarried/unpartnered (OR = 0.77, 95% CI: 0.73–0.81), 36% lower odds of isolation from family members (OR = 0.64, 95% CI: 0.51–0.71), 43% lower odds of isolation from in-person visits (OR = 0.57, 95% CI: 0.51–0.63) and isolation from religious attendance (OR = 0.57, 95% CI: 0.49–0.66), and 36% lower odds of isolation from club participation (OR = 0.64, 95% CI: 0.57–0.72).

On the other hand, the presence of physical disorder in the neighborhood was significantly associated with increased risk of individual isolation domains in being unmarried/unpartnered, no in-person visits, and no club participation. In particular, compared to respondents living in neighborhoods without any signs of physical disorder, the presence of physical disorder was associated with 1.6 times higher odds of being unmarried/unpartnered (95% CI: 1.47, 1.77), 1.4 times higher odds of no in-person visits (95% CI: 1.17, 1.64), and 1.2 times higher odds of no club participation (95% CI: 1.00, 1.45). We observed no longitudinal effects of neighborhood physical disorder on overall social isolation and isolation from family, friends, and religious attendance. Supplementary analyses among non-proxy respondents (Supplementary Table 4) and using 1-year lagged neighborhood measures (Supplementary Table 5) yielded results that conformed to the main analyses.

### Hypothesis 2

**Fig. 3:**
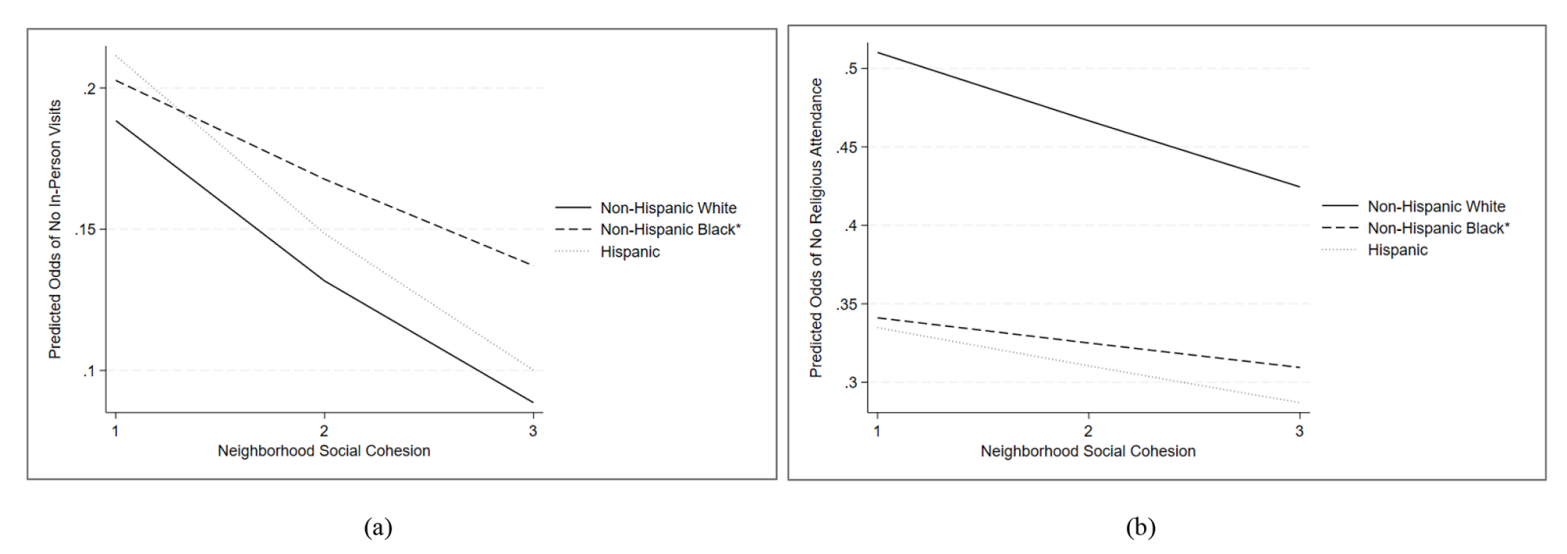
Significant interactions between neighborhood social cohesion and race/ethnicity in predicting the odds of (**a**) isolation from in-person visits with family and friends and (**b**) isolation from religious attendance, adjusting for socio-demographic, health controls, and receiving caregiving. Results are based on Supplementary Table 2. Reference group = White. *p < 0.05

Race/ethnicity significantly moderated the effects of neighborhood social cohesion on in-person visits with family/friends and religious attendance, as plotted in Fig. 3 (based on Supplementary Table 2). We included the “other race” group to maintain a larger sample size, but did not plot findings for this group due to difficulty in generalizing the findings to a specific racial/ethnic group. Regarding the interactive findings for isolation from in-person family and friend visits, while we observed decreased odds of no in-person visits associated with higher levels of social cohesion among all racial and ethnic groups, the odds decreased at a significantly slower rate among older Black adults compared to older White adults. With respect to the interactive findings for isolation from religious attendance, older Black adults had constantly lower odds of isolation from religious attendance compared to older White adults regardless of the level of neighborhood social cohesion. For older White adults, the odds of no religious attendance decreased as perceptions of social cohesion increased.

**Fig. 4:**
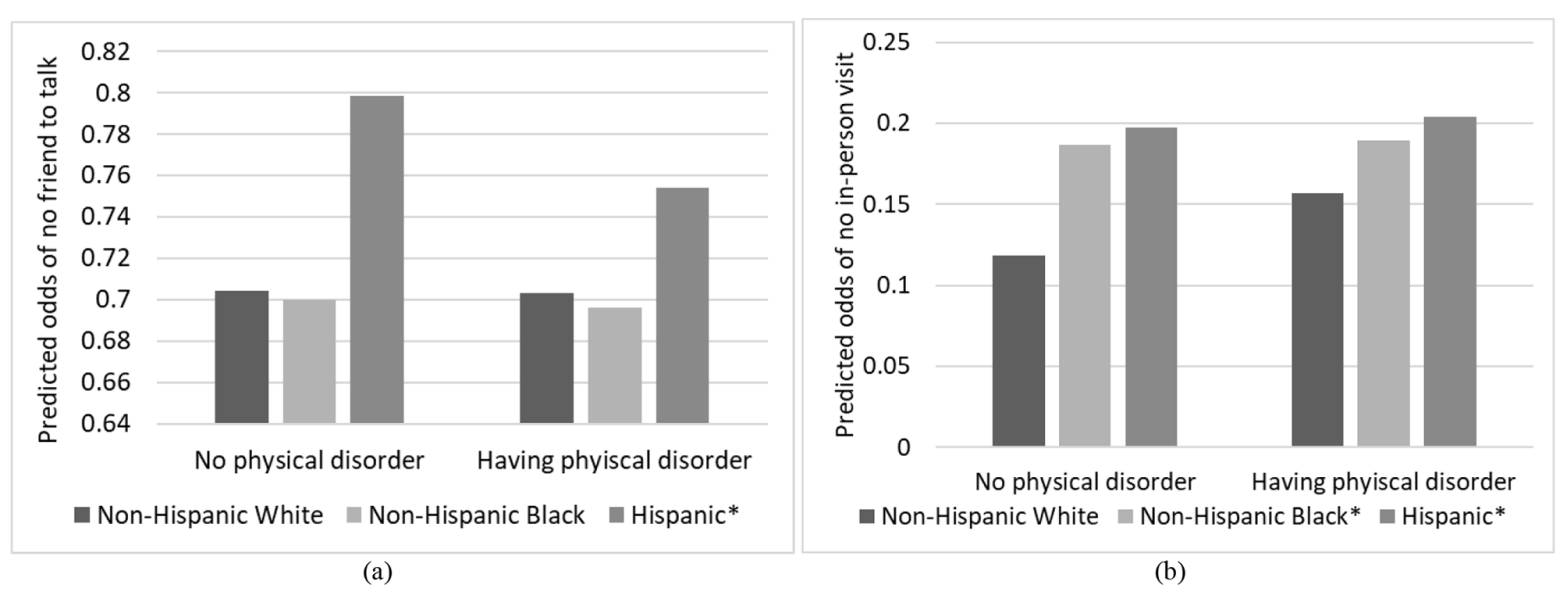
Significant interactions between neighborhood physical disorder and race/ethnicity in predicting odds of (**a**) no friend to talk to and (**b**) no in-person visits with family or friends, adjusting for socio-demographic, health controls and receiving caregiving. Results are based on the Supplementary Table 3. Reference group = White. *p < 0.05

In addition, race/ethnicity significantly moderated the effects of neighborhood physical disorder on having friends to talk to and in-person visits, which were plotted in Fig. 4 (based on Supplementary Table 3). The significant interaction for having no friends indicated that among older adults living in neighborhoods with any signs of physical disorder, older Hispanic adults had decreased odds of having no friends, while no differences in isolation from friends by physical disorder status were found among older White adults. Turning to the significant interaction for in-person visits, the findings revealed that older Black and Hispanic adults reported no difference in the odds of isolation from in-person visits between living in neighborhood with and without physical disorder, whereas older White adults had higher odds of isolation from in-person visits when living in neighborhoods with any signs of physical disorder.

## Discussion

Using 11-year data from a national survey study among older Medicare beneficiaries, our study seeks to understand the longitudinal associations between neighborhood characteristics and social isolation, as well as racial and ethnic differences in the neighborhood-isolation associations. Two primary findings emerged. First, neighborhood characteristics are associated with both overall social isolation and some individual domains of isolation. Specifically, a greater level of neighborhood social cohesion is related to lower odds of overall social isolation and all individual domains of isolation except isolation from friends. On the contrary, visible signs of physical disorder in one’s neighborhood are associated with increased odds of overall social isolation and all domains of isolation except isolation from family, friends, and religious attendance. Second, race and ethnicity demonstrate moderating effects on the association between neighborhood characteristics and social isolation. That is, the magnitude of the association between neighborhood and some domains of isolation (i.e., isolation from family/friends and church attendance) vary across racial and ethnic groups. Given the limited research on environmental factors of social isolation among older adults [11, 26], our study expands the evidence on longitudinal effects of neighborhood-level factors on social isolation among older adults.

The findings largely supported Hypothesis 1 regarding the longitudinal effects of neighborhood characteristics on social isolation. Consistent with previous evidence [19, 21], we found that stronger neighborhood social cohesion may protect against both overall and individual domains of social isolation over time. Stronger social cohesion in the neighborhood may foster older adults’ connections with their neighbors and facilitate participation in community activities at church, organizations, and clubs. In related research, evidence suggests that greater neighborhood social cohesion is associated with greater likelihood of visiting family and friends in person [21]. Strong social cohesion in the neighborhood may be indicative of trusting relationships and a sense of safety in the community that encourages older adults to engage in social interactions with network members.

On the other hand, the presence of physical disorder is related to increased odds of social isolation. Visual signs of physical decline and decay in the neighborhood can be stress-inducing and lead to a sense of fear [51]. For example, litter and trash in the neighborhood can be a cue of physical hazards that deter older adults from going outdoors [17]. As such, older adults may have reduced willingness to visit family and friends or participate in organized activities outside of the home. However, signs of physical disorder appear to have limited impact on older adults’ participation in religious services. Given that religious involvement is important to late-life mental health and well-being among older adults with high levels of religiosity [52], it is possible that some older adults consider religious services as an essential source of coping, and actively attend church activities regardless of neighborhood conditions. In alignment with the Social Disorganization Theory which suggests that living in a neighborhood characterized by perceived disorders can damage health [12, 14], our findings indicate that perceptions of neighborhood disadvantages can influence older adults’ behaviors and are linked to increased risk of social isolation.

Additionally, our study reveals significant racial and ethnic differences in social isolation among older adults. While older White adults may have lower odds of overall isolation and individual isolation domains than older Black adults, Black and Hispanic adults are less likely to be isolated from religious attendance than White individuals. Our study echoes the mixed findings on racial differences in social isolation. Earlier research shows that older Black adults are more likely to be socially isolated than White adults [53], whereas more recent findings indicate that White adults are at higher odds of severe social isolation compared to older Black and Hispanic adults [28]. By comparing individual domains of isolation across race and ethnicity, our study indicates the need to examine racial differences in social isolation within larger context such as neighborhood factors.

Our findings partially confirmed Hypothesis 2 regarding the moderating effects of race and ethnicity on the neighborhood-isolation associations. Interestingly, our findings indicate that greater neighborhood social cohesion is associated with lower odds of isolation from religious services among older White adults, but older Black adults have consistently lower odds of isolation from religious services regardless of the levels of neighborhood social cohesion. Bivariate findings show that older Black adults experience less isolation from religious attendance compared to older White adults. Findings show that older Black adults tend to live in neighborhood with lower social cohesion than White adults. Religion is a significant and meaningful aspect of many older Black adults’ lives. Religion is intricately woven into the social, cultural, and ethnic fabric of Black American communities [54]. Religious traditions rooted in liberation and defiance theology offer distinct resources for coping with discrimination and other chronic stressors experienced by this community [55]. Accordingly, older Black adults are the most religious demographic group in the U.S., reporting higher rates of service attendance and other forms of organizational and non-organizational religious participation than their younger and White counterparts [56–58]. In fact, previous research reveals that, compared to older White adults, older Black adults are more likely to gain health-related benefits through religious participation [58]. Given the centrality of religion in older Black Americans’ lives, the lack of neighborhood social cohesion may not be significant enough of a barrier to prevent older Black adults from attending religious services. Taken together, service attendance may provide a level of social integration that serves as an alternative source of social cohesion among older Black adults living in neighborhoods with low cohesion.

Neighborhood physical disorder demonstrated differing effects on social isolation across racial and ethnic groups. Specifically, any signs of neighborhood physical disorder are associated with lower odds of isolation from friends among older Hispanic adults, but this association is non-significant among older White adults. Prior findings indicate that Hispanic individuals’ support networks are characterized by greater geographic proximity [59]. Therefore, it is possible that older Hispanic adults in neighborhood with physical disorder may also live close to or even in the same neighborhoods as their friends, who constitute a strong support network. From the perspective of resource mobilization [60], individuals in stressful situations, such as living in deteriorating and unsafe neighborhoods, may also actively seek coping resources and enhance connections with their friends, which further prevents social isolation. Additionally, our findings show that older Hispanic adults are significantly more likely to reside in neighborhoods characterized by high perceived disorder compared to White adults. It is possible that Hispanic individuals tend to live in ethnic enclaves, which can sometimes be disinvested, and thus experience more disadvantages. While racial and ethnic segregation can have adverse impact on a wide range of psychosocial outcomes, one unintended consequence is that individuals living in segregated neighborhoods are more likely to live near family and other people who share similar culture [61]. This may provide social ties and social connectedness that reduce the risk of isolation.

Additionally, older Black adults reported similar odds of no in-person visits regardless of neighborhood physical disorder, while older White adults are more likely to report no in-person visits when living in neighborhoods with signs of physical disorder. Relatedly, a recent study has noted that subjective neighborhood perceptions have limited influence on social isolation among older Black adults [26]. Our findings that older Black adults’ in-person visits with family and friends are not influenced by neighborhood physical disorder may reflect that family and friend networks are important sources of emotional support and coping for this group regardless of environmental context. Another consideration of the finding relates to the historical racial segregation of Black populations. Historically and contemporaneously, Black individuals have been forced to living in racially segregated neighborhoods that are often characterized by social, economic, and political disinvestment [16]. Given the principle of homophily in social networks (i.e., people tend to have family and friends that are similar in sociodemographic, behavioral, and intrapersonal characteristics) [62], family and friends may live in similar neighborhoods and the presence of deterioration in the built environment may not discourage in-person visits.

Racial and ethnic differences in the association between neighborhood factors and social isolation may also speak to differential exposure as well as differential reactivity. Our findings, consistent with previous evidence [38], suggest that older Black and Hispanic individuals are more likely to reside in neighborhoods with lower level of social cohesion and higher levels of physical disorder compared to White individuals. The differential exposure to neighborhood protective and risk factors may place Black and Hispanic individuals at higher risk of social isolation. On the other hand, despite lower level of social cohesion, Black and Hispanic adults are less likely to experience isolation from religious attendance, suggesting differential reactions and coping method (i.e., church attendance) in response to stressful neighborhood environment.

### Limitations

The findings should be interpreted considering the limitations. First, the measures in our study are all based on participant-reported or interviewer-observed data, rendering the findings subject to recall bias and social desirability. The reliance on subjective data with a lack of objective census tract-level data can hinder a more comprehensive understanding of the pathways linking neighborhood factors to isolation status. However, research on neighborhoods and health suggests that subjective perceptions of neighborhoods appear to have a stronger effects on health than objective measures [63]. Prior evidence also indicates that self-reported perceptions of neighborhood conditions are a useful alternative to capture neighborhood characteristics when objective measures are not available [64]. The utilization of a binary measure of social isolation does not fully capture the complexity, severity and multi-dimensionality of this concept, limiting the generalization of the study findings. Second, since the study data are observational, we cannot make causal inferences regarding the neighborhood-isolation association. It is possible that older adults feeling isolated are more likely to perceive their neighborhood environment as negative. The longitudinal association between neighborhood factors and social isolation may be influenced by the baseline wave’s cross-sectional association carrying over to the subsequent wave. Another limitation relates to the use of culturally valid measures of neighborhood perceptions. Given older Black and Hispanic adults’ distinct social and cultural experiences, neighborhood perceptions may carry non-equivalent meaning across different racial and ethnic groups. Additional measurement studies on neighborhood perceptions focusing on older Black and Hispanic adults are needed to further examine the pathway linking neighborhood to social isolation.

## Conclusions

Social isolation remains a critical public health concern that affects millions of older Americans lacking social connections in one or multiple domains. The neighborhood represents an important environmental determinant of health that may be linked to long-term social isolation. Community-level efforts to improve trust and social cohesion within the neighborhood and to develop physical neighborhood features that encourage communal gatherings may help reduce social isolation in older adults.

Evidence from our longitudinal findings can also inform neighborhood-level interventions aiming at reducing social isolation among older adults. Interventions providing structured networking sessions for older adults in the neighborhood have been shown to increase perceived social support and sense of community. For example, an existing intervention piloted among Hispanic individuals focused on increasing social interactions among neighbors by facilitating discussions on local community needs [65]. Similarly, a culturally tailored intervention was developed to promote mental health among older Black adults by focusing on trusting social environment [66]. However, neighborhood-level interventions on reducing social isolation are currently limited. Our findings suggest that there is a need to study the long-term effects of such interventions on social isolation.

Additionally, our findings suggest that social isolation among Black and Hispanic adults are less likely to be influenced by neighborhood perceptions than older White adults. Further research is needed to clarify the pathways linking contextual factors to social isolation among older Black and Hispanic populations and to better understand how unique social processes within the neighborhood affect health among the populations.

## Electronic supplementary material

Below is the link to the electronic supplementary material.

Supplementary Material 1

## Data Availability

The dataset supporting the conclusions of this article is publicly available at www.nhats.org.
