# Supplementary material for: Neighborhood social cohesion and physical disorder in relation to social isolation in older adults: racial and ethnic differences
Source: BMC Public Health. 2024 Sep 20;24:2574. doi: 10.1186/s12889-024-20112-9 (PMC11414110; doi:10.1186/s12889-024-20112-9)
Supplement: Supplementary file 1 — Supplementary Material 1 [file 12889_2024_20112_MOESM1_ESM.pdf]

Supplementary Table 1. Main effects of Neighborhood Characteristics Predicting Social Isolation from Mixed-Effects Logistic Regressions

Predictors	Overall isolation	Unmarried/ unpartnered	No family to talk to	No friends to talk to	No in-person visit with family or friends	No religious attendance	No club participation
	OR (95% CI)						
Neighborhood social cohesion	0.54*** (0.48, 0.60)	0.77*** (0.73, 0.81)	0.64*** (0.56, 0.71)	0.93 (0.82, 1.05)	0.57*** (0.51, 0.63)	0.57*** (0.49, 0.66)	0.64*** (0.57, 0.72)
Neighborhood physical disorder (ref=no)	1.18 (0.98, 1.42)	1.61*** (1.47, 1.77)	1.14 (0.93, 1.40)	0.92 (0.74, 1.14)	1.38*** (1.17, 1.64)	1.19 (0.97, 1.46)	1.21* (1.00, 1.45)
Race/ethnicity (ref=White)							
Non-Hispanic Black	1.75*** (1.33, 2.30)	2.13*** (1.97, 2.31)	2.37*** (1.78, 3.14)	0.96 (0.73, 1.26)	1.61*** (1.34, 1.94)	0.12*** (0.08, 0.17)	1.38** (1.09, 1.75)
Hispanic	0.92 (0.59, 1.44)	0.96 (0.83, 1.10)	0.85 (0.57, 1.27)	2.95*** (1.64, 5.29)	1.22 (0.87, 1.72)	0.08*** (0.05, 0.15)	2.69*** (1.74, 4.15)
Other	2.21** (1.40, 3.49)	0.95 (0.80, 1.13)	1.20 (0.58, 2.49)	2.35** (1.31, 4.21)	1.98** (1.25, 3.14)	0.45* (0.22, 0.94)	2.65*** (1.61, 4.36)
**Model statistics**							
$\chi 2$ (df)	4654***	5959***	10276***	2900***	6357***	1960***	11346***
# of persons	7,305	7,303	7,291	7,139	7,304	7,304	7,305
# of person-years	34,066	34,056	33,990	32,898	34,050	34,048	34,044

Note. All models controlled for age, gender, education, dementia status, count of chronic conditions, depressive symptoms, receiving caregiving, proxy status, and survey round. OR = odds ratio. CI = confidence interval. Ref=reference group. NHATS-provided sampling weights and design factors were incorporated to adjust for complex sample design and generate weighted estimates.

*p < 0.05; **p < 0.01; ***p < 0.001

Supplementary Table 2. Interaction Effects Between Race/Ethnicity and Neighborhood Social Cohesion in Predicting Social Isolation

Predictors	Overall isolation	Unmarried/ unpartnered	No family to talk to	No friends to talk to	No in-person visit with family or friends	No religious attendance	No club participation
	OR (95% CI)						
Neighborhood social cohesion × race/ethnicity (ref=white)							
Neighborhood social cohesion × Black	1.27 (0.96, 1.67)	0.99 (0.88, 1.12)	1.09 (0.81, 1.46)	1.03 (0.78, 1.36)	1.31* (1.04, 1.65)	1.42* (1.04, 1.92)	0.99 (0.74, 1.33)
Neighborhood social cohesion × Hispanic	1.09 (0.68, 1.74)	0.94 (0.78, 1.14)	1.14 (0.75, 1.75)	1.12 (0.67, 1.88)	0.98 (0.70, 1.38)	1.22 (0.73, 2.02)	1.38 (0.90, 2.11)
Neighborhood social cohesion × Other	1.31 (0.76, 2.26)	1.08 (0.78, 1.50)	1.38 (0.64, 2.97)	1.25 (0.71, 2.19)	1.23 (0.79, 1.92)	1.32 (0.55, 3.17)	1.14 (0.71, 1.84)
Neighborhood Social Cohesion	0.50*** (0.44, 0.57)	0.77*** (0.73, 0.82)	0.61*** (0.53, 0.71)	0.92 (0.80, 1.05)	0.54*** (0.48, 0.62)	0.54*** (0.45, 0.63)	0.62*** (0.55, 0.71)
Race/ethnicity (ref=White)							
Non-Hispanic Black	1.05 (0.54, 2.06)	2.17*** (1.62, 2.90)	1.93* (1.00, 3.71)	0.89 (0.44, 1.79)	0.87 (0.48, 1.57)	0.05*** (0.02, 0.11)	1.41 (0.69, 2.86)
Hispanic	1.07 (0.35, 3.25)	1.10 (0.70, 1.75)	0.63 (0.24, 1.68)	2.29 (0.64, 8.19)	1.25 (0.52, 3.04)	0.06*** (0.02, 0.19)	1.30 (0.47, 3.55)
Other	1.38 (0.37, 5.19)	0.79 (0.36, 1.72)	0.57 (0.09, 3.54)	1.39 (0.33, 5.81)	1.23 (0.43, 3.53)	0.23 (0.02, 2.12)	1.92 (0.55, 6.67)
# of persons	7,305	7,303	7,291	7,139	7,304	7,304	7,305
# of person-years	34,066	34,056	33,990	32,898	34,050	34,048	34,044

Note. All models controlled for age, gender, education, dementia status, count of chronic conditions, depressive symptoms, , receiving caregiving, proxy status, and survey round. OR = odds ratio. CI = confidence interval. Ref=reference group. NHATS-provided sampling weights and design factors were incorporated to adjust for complex sample design and generate weighted estimates.
*p < 0.05; **p < 0.01; ***p < 0.001

Supplementary Table 3. Interaction Effects Between Race/ethnicity and Neighborhood Physical Disorder in Predicting Social Isolation

Predictors	Overall isolation	Unmarried/ unpartnered	No family to talk to	No friends to talk to	No in-person visit with family or friends	No religious attendance	No club participation
	OR (95% CI)						
Neighborhood physical disorder × Race/ethnicity (ref=white)							
Neighborhood physical disorder × Black	0.81 (0.55, 1.20)	0.88 (0.72, 1.07)	1.04 (0.65, 1.66)	0.97 (0.65, 1.44)	0.65** (0.51, 0.83)	0.88 (0.56, 1.38)	0.99 (0.71, 1.39)
Neighborhood physical disorder × Hispanic	0.86 (0.49, 1.50)	0.84 (0.62, 1.12)	1.18 (0.59, 2.39)	0.55* (0.32, 0.96)	0.68* (0.49, 0.95)	1.20 (0.60, 2.37)	0.68 (0.42, 1.09)
Neighborhood physical disorder × other	1.04 (0.47, 2.29)	1.08 (0.65, 7.80)	2.04 (0.86, 4.83)	0.88 (0.28, 2.81)	0.83 (0.46, 1.51)	1.28 (0.45, 3.63)	0.32** (0.16, 0.64)
Neighborhood physical disorder (ref=no)	1.26* (0.99, 1.59)	1.72*** (1.51, 1.96)	1.07 (0.80, 1.42)	0.98 (0.73, 1.33)	1.57*** (1.32, 1.87)	1.16 (0.92, 1.48)	1.32* (1.04, 1.69)
Race/ethnicity (ref=White)							
Non-Hispanic Black	1.90*** (1.44, 2.52)	2.17*** (2.00, 2.36)	2.37*** (1.77, 3.19)	0.95 (0.72, 1.25)	2.12*** (1.86, 2.43)	0.12*** (0.08, 0.18)	1.36** (1.06, 1.74)
Hispanic	1.35 (0.85, 2.14)	0.99 (0.85, 1.15)	0.82 (0.53, 1.28)	3.38*** (1.84, 6.22)	2.34 (1.86, 2.93)	0.08*** (0.04, 0.15)	2.87*** (1.82, 4.51)
Other	2.59*** (1.71, 3.92)	0.94 (0.78, 1.13)	1.09 (0.52, 2.30)	2.37** (1.31, 4.28)	2.39 (1.75, 3.26)	0.44 (0.22, 0.90)	3.00*** (1.79, 5.02)
# of persons	7,305	7,303	7,291	7,139	7,304	7,304	7,305
# of person-years	34,066	34,056	33,990	32,898	34,050	34,048	34,044

Note. All models controlled for age, gender, education, dementia status, count of chronic conditions, depressive symptoms, receiving caregiving, proxy status, and survey round. OR = odds ratio. CI = confidence interval. Ref=reference group. NHATS-provided sampling weights and design factors were incorporated to adjust for complex sample design and generate weighted estimates.

*p < 0.05; **p < 0.01; ***p < 0.001

Supplementary Table 4. Results from Mixed-Effects Models Conducted Among Non-Proxy Respondents

Predictors	Overall isolation	Unmarried/ unpartnered	No family to talk to	No friends to talk to	No in-person visit with family or friends	No religious attendance	No club participation
	OR (95% CI)						
Neighborhood social cohesion	0.51*** (0.46, 0.58)	0.76*** (0.72, 0.80)	0.63*** (0.56, 0.71)	0.93 (0.82, 1.05)	0.56*** (0.50, 0.64)	0.57*** (0.48, 0.66)	0.64*** (0.56, 0.72)
Neighborhood physical disorder (ref=no)	1.16 (0.96, 1.41)	1.61*** (1.47, 1.77)	1.14 (0.93, 1.39)	0.92 (0.74, 1.14)	1.34** (1.12, 1.62)	1.18 (0.95, 1.46)	1.21 (0.99, 1.46)
Race/ethnicity (ref=White)							
Non-Hispanic Black	1.87*** (1.41, 2.47)	2.12*** (1.95, 2.30)	2.44*** (1.84, 3.26)	0.97 (0.74, 1.27)	1.74*** (1.43, 2.12)	0.76*** (0.03, 0.18)	1.35* (1.07, 1.69)
Hispanic	1.33 (0.84, 2.12)	0.93 (0.80, 1.08)	0.82 (0.54, 1.24)	2.96*** (1.65, 5.32)	1.37 (0.94, 1.99)	0.04*** (0.01, 0.14)	2.66*** (1.72, 4.12)
Other	2.83*** (1.79, 4.48)	1.02 (0.84, 1.22)	1.26 (0.61, 2.59)	2.32** (1.29, 4.16)	2.18** (1.31, 3.64)	0.62 (0.26, 1.48)	2.34** (1.35, 4.04)
# of persons	6,868	6,868	6,853	6,676	6,867	6,868	6,868
# of person-years	32,053	32,044	31,977	30,885	32,039	32,041	32,034

Note. All models controlled for age, gender, education, dementia status, count of chronic conditions, depressive symptoms, , receiving caregiving, proxy status, and survey round. OR = odds ratio. CI = confidence interval. Ref=reference group.
*p < 0.05; **p < 0.01; ***p < 0.001

Supplementary Table 5. Results from Mixed-Effects Models Using 1-Year Lagged Neighborhood Factors predicting Social Isolation

Predictors (Round t-1)	Overall isolation (Round t)	Unmarried/ unpartnered (Round t)	No family to talk to (Round t)	No friends to talk to (Round t)	No in-person visit with family or friends (Round t)	No religious attendance (Round t)	No club participation (Round t)
	OR (95% CI)						
Neighborhood social cohesion	0.68*** (0.61, 0.77)	0.77*** (0.72, 0.81)	0.88* (0.79, 0.97)	0.90 (0.78, 1.04)	0.74*** (0.69, 0.80)	0.65*** (0.57, 0.74)	0.74*** (0.65, 0.84)
Neighborhood physical disorder (ref=no)	1.55*** (1.33, 1.80)	1.56*** (1.41, 1.73)	1.07 (0.91, 1.26)	0.91 (0.78, 1.08)	1.28*** (1.14, 1.43)	1.16 (0.94, 1.44)	1.51*** (1.20, 1.90)
Race/ethnicity (ref=White)							
Non-Hispanic Black	1.64*** (1.30, 2.05)	2.15*** (1.96, 2.35)	1.59*** (1.31, 1.93)	0.99 (0.72, 1.36)	1.84*** (1.62, 2.10)	0.24*** (0.18, 0.32)	1.55*** (1.22, 1.97)
Hispanic	1.41 (0.94, 2.13)	0.99 (1.96, 2.35)	0.94 (0.68, 1.31)	2.35** (1.32, 4.19)	2.25*** (1.81, 2.80)	0.18*** (0.10, 0.32)	3.18*** (2.01, 5.01)
Other	2.35*** (1.59, 3.47)	1.00 (0.82, 1.22)	1.42 (0.86, 2.35)	2.00* (1.05, 3.79)	2.18*** (1.60, 2.97)	0.65 (0.33, 1.28)	3.21*** (1.81, 5.71)
# of persons	6,265	6,054	6,260	6,210	5,914	5,873	5,873
# of person-years	29,927	27,921	29,903	29,334	28,365	27,894	27,894

Note. All models controlled for age, gender, education, dementia status, count of chronic conditions, depressive symptoms, , receiving caregiving, proxy status, and survey round. OR = odds ratio. CI = confidence interval. Ref=reference group. Lagged neighborhood factors at Round t-1 were used to predict social isolation at Round t.

*p < 0.05; **p < 0.01; ***p < 0.001

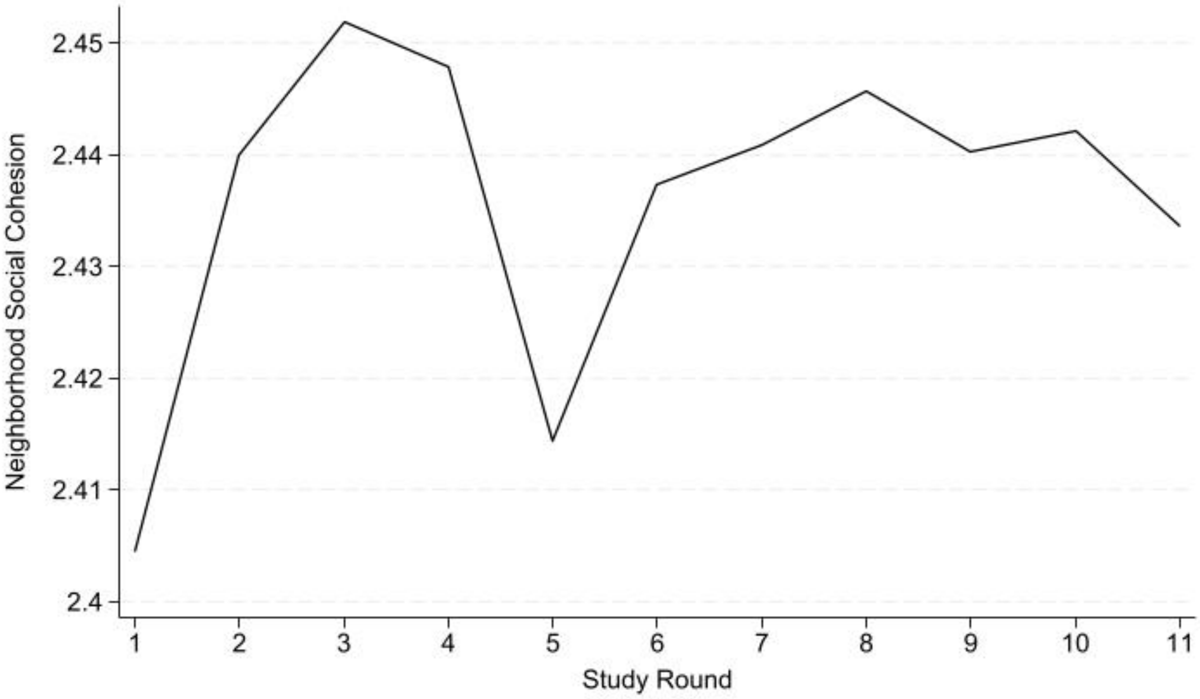

Supplementary Figure 1. Means of neighborhood social cohesion over 11 years.

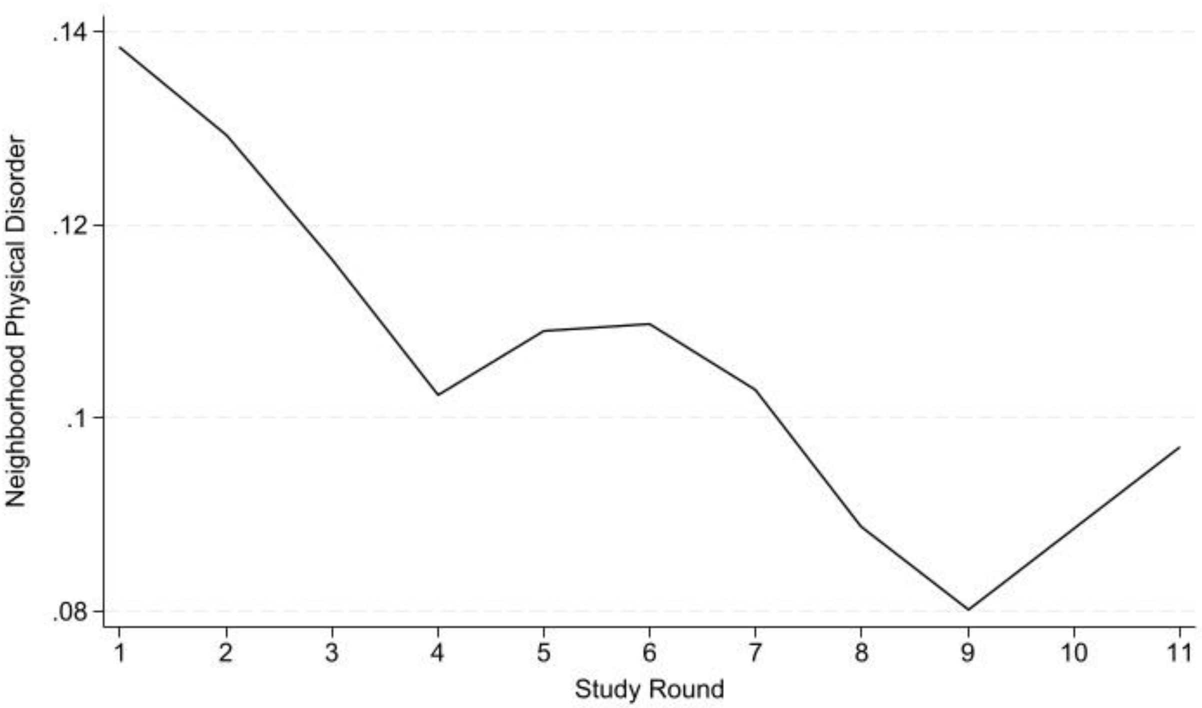

Supplementary Figure 2. Means of neighborhood physical disorder over 11 years.

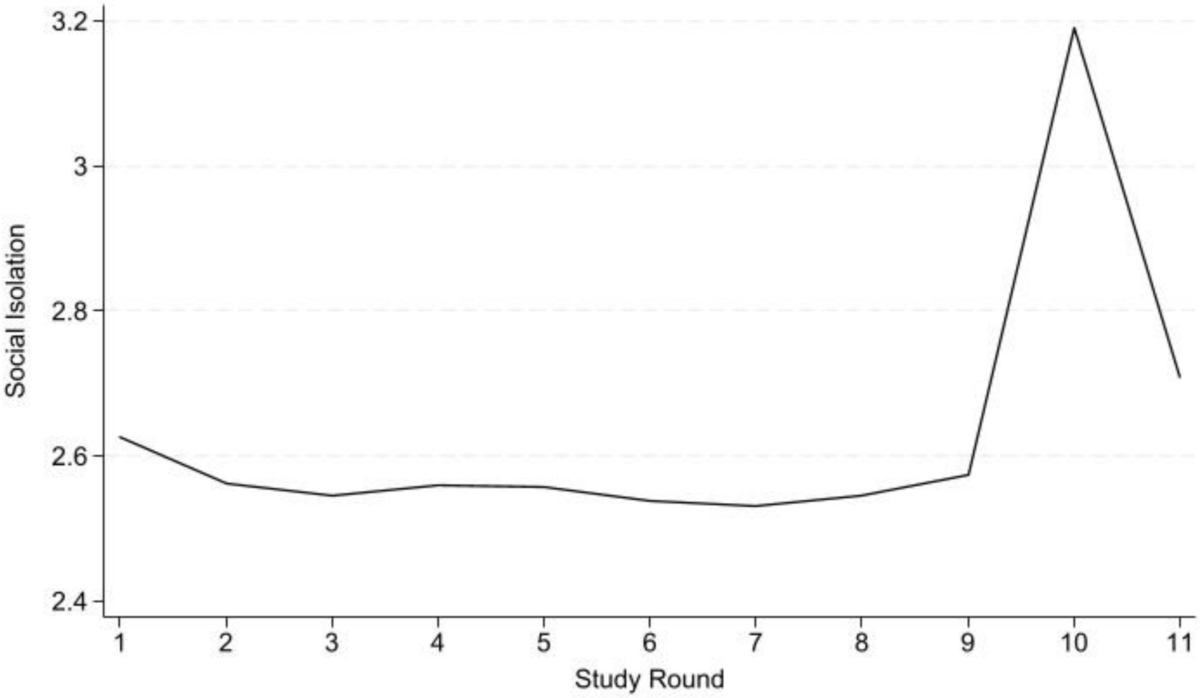

Supplementary Figure 3. Means of social isolation over 11 years.
